# FAIRe Gesundheitsdaten im nationalen und internationalen Datenraum

**DOI:** 10.1007/s00103-024-03884-8

**Published:** 2024-05-15

**Authors:** Dagmar Waltemath, Oya Beyan, Katrin Crameri, Angela Dedié, Kerstin Gierend, Petra Gröber, Esther Thea Inau, Lea Michaelis, Ines Reinecke, Martin Sedlmayr, Sylvia Thun, Dagmar Krefting

**Affiliations:** 1 Abteilung Medizininformatik, Institut für Community Medicine, Walther-Rathenau-Straße 48, 17475 Greifswald, Deutschland; 2 Medizinische Fakultät und Uniklinik Köln, Institut für Biomedizininformatik, Universität zu Köln, Köln, Deutschland; 3 Schweizerisches Institut für Bioinformatik, Personalisierte Gesundheitsinformatik, Basel, Schweiz; 4 Deutsches Zentrum für Diabetesforschung (DZD), Geschäftsstelle am Helmholtz Zentrum München, München, Deutschland; 5 Abteilung für Biomedizinische Informatik am Zentrum für Präventivmedizin und Digitale Gesundheit (CPD), Medizinische Fakultät Mannheim der Universität Heidelberg, Mannheim, Deutschland; 6 Datenintegrationszentrum Universitätsmedizin Rostock, Rostock, Deutschland; 7 Datenintegrationszentrum, Zentrum für Medizinische Informatik, Universitätsklinikum Carl Gustav Carus Dresden, Dresden, Deutschland; 8 Institut für Medizinische Informatik und Biometrie, Med. Fakultät Carl Gustav Carus, TU Dresden, Dresden, Deutschland; 9 Berliner Institut für Gesundheitsforschung in der Charité – Universitätsmedizin Berlin, Berlin, Deutschland; 10 Institut für Medizinische Informatik, Universitätsmedizin Göttingen und Deutsches Zentrum für Herz-Kreislauf-Forschung, Partner Site Göttingen, Göttingen, Deutschland

**Keywords:** Datenmanagement, Interoperabilität, Datenintegration, IT-Infrastruktur, Netzwerk Universitätsmedizin, Data management, Interoperability, Data integration, IT infrastructure, German Network University Medicine

## Abstract

Gesundheitsdaten haben in der heutigen datenorientierten Welt einen hohen Stellenwert. Durch automatisierte Verarbeitung können z. B. Prozesse im Gesundheitswesen optimiert und klinische Entscheidungen unterstützt werden. Dabei sind Aussagekraft, Qualität und Vertrauenswürdigkeit der Daten wichtig. Nur so kann garantiert werden, dass die Daten sinnvoll nachgenutzt werden können.

Konkrete Anforderungen an die Beschreibung und Kodierung von Daten werden in den FAIR-Prinzipien beschrieben. Verschiedene nationale Forschungsverbünde und Infrastrukturprojekte im Gesundheitswesen haben sich bereits klar zu den FAIR-Prinzipien positioniert: Sowohl die Infrastrukturen der Medizininformatik-Initiative als auch des Netzwerks Universitätsmedizin operieren explizit auf Basis der FAIR-Prinzipien, ebenso die Nationale Forschungsdateninfrastruktur für personenbezogene Gesundheitsdaten oder das Deutsche Zentrum für Diabetesforschung.

Um eine FAIRe Ressource bereitzustellen, sollte zuerst in einem Assessment der FAIRness-Grad festgestellt werden und danach die Priorisierung für Verbesserungsschritte erfolgen (FAIRification). Seit 2016 wurden zahlreiche Werkzeuge und Richtlinien für beide Schritte entwickelt, basierend auf den unterschiedlichen, domänenspezifischen Interpretationen der FAIR-Prinzipien.

Auch die europäischen Nachbarländer haben in die Entwicklung eines nationalen Rahmens für semantische Interoperabilität im Kontext der FAIR-Prinzipien investiert. So wurden Konzepte für eine umfassende Datenanreicherung entwickelt, um die Datenanalyse beispielsweise im Europäischen Gesundheitsdatenraum oder über das Netzwerk der Observational Health Data Sciences and Informatics zu vereinfachen. In Kooperation mit internationalen Projekten, wie z. B. der European Open Science Cloud, wurden strukturierte FAIRification-Maßnahmen für Gesundheitsdatensätze entwickelt.

## Abstract (en)

Health data are extremely important in today’s data-driven world. Through automation, healthcare processes can be optimized, and clinical decisions can be supported. For any reuse of data, the quality, validity, and trustworthiness of data are essential, and it is the only way to guarantee that data can be reused sensibly. Specific requirements for the description and coding of reusable data are defined in the FAIR guiding principles for data stewardship. Various national research associations and infrastructure projects in the German healthcare sector have already clearly positioned themselves on the FAIR principles: both the infrastructures of the Medical Informatics Initiative and the University Medicine Network operate explicitly on the basis of the FAIR principles, as do the National Research Data Infrastructure for Personal Health Data and the German Center for Diabetes Research.

To ensure that a resource complies with the FAIR principles, the degree of FAIRness should first be determined (so-called FAIR assessment), followed by the prioritization for improvement steps (so-called FAIRification). Since 2016, a set of tools and guidelines have been developed for both steps, based on the different, domain-specific interpretations of the FAIR principles.

Neighboring European countries have also invested in the development of a national framework for semantic interoperability in the context of the FAIR (Findable, Accessible, Interoperable, Reusable) principles. Concepts for comprehensive data enrichment were developed to simplify data analysis, for example, in the European Health Data Space or via the Observational Health Data Sciences and Informatics network. With the support of the European Open Science Cloud, among others, structured FAIRification measures have already been taken for German health datasets.

## Hintergrund

Gesundheitsdaten haben in der heutigen datenorientierten Welt einen hohen Stellenwert. Durch automatisierte Verarbeitung können beispielsweise Prozesse im Gesundheitswesen optimiert und klinische Entscheidungen unterstützt werden. Dabei ist es wichtig, dass die Daten aussagekräftig, von hoher Qualität und vertrauenswürdig sind. Nur so kann eine sinnvolle automatische Verarbeitung, aber auch Nachnutzung garantiert werden. Die Erhebung von Primärdaten – sei es im Behandlungs- oder Studienkontext – ist ein aufwendiger, kosten- und zeitintensiver Prozess, der verteilt über verschiedene Anwendungssysteme und auch Klinik- und Landesgrenzen hinweg stattfindet. Sekundärdatennutzung wiederum macht moderne medizinische Forschung, die auf große Datensätze angewiesen ist, erst möglich, beispielsweise für das Trainieren künstlicher Intelligenz (KI).

Der Datenbereitstellung und -nachnutzung widmen sich verschiedene Initiativen in Deutschland. Neben der Medizininformatik-Initiative (MII; [1]), welche fachübergreifend strukturierte Daten aus den Krankenhausinformationssystemen in den lokalen Datenintegrationszentren der Universitätskliniken verfügbar macht, gibt es zahlreiche fachspezifische Initiativen. Beispiele sind das Radiologienetzwerk RACOON (RAdiological COOperative Network; [2]), die COMPASS(„coordination on mobile pandemic apps best practice and solution sharing“; [3])-Plattform^1^ für den Einsatz von Gesundheits-Apps in Pandemiesituationen, das Notaufnahmeregister AKTIN (Aktionsbündnis zur Verbesserung der Kommunikations- und Informationstechnologie in der Intensiv- und Notfallmedizin; [4]) im Rahmen des Netzwerks Universitätsmedizin (NUM; [5]) oder RADARplus (Routine Anonymized Data for Advanced Health Services Research; [6]) für die Nutzung von Daten aus Hausarztpraxen. Das Deutsche Zentrum für Herz- und Kreislauferkrankungen (DZHK) baut für die Sekundärnutzung von Studiendaten seit 2012 die sogenannte Heart Bank auf, deren Plattform seit 2020 auch für die nationalen COVID-19-Kohorten genutzt und nun als Infrastruktur im NUM weiterentwickelt wird [7, 8].

Die gemeinsame Nutzung von Gesundheitsdaten für die Forschung gelingt dann, wenn die Daten und die beteiligten datenhaltenden Systeme interoperabel sind, d. h. von verschiedenen IT-Systemen und Datenbanken verarbeitet und interpretiert werden können. Dabei ist es wichtig, dass auch Bearbeitungsprozesse und die Herkunft der Daten dokumentiert werden. Dies wird als Provenienz bezeichnet. Provenienz schafft dadurch einen Mehrwert, indem sie Wiederverwendungsmöglichkeiten, aber auch Grenzen aufzeigt und Vertrauen in die Daten schafft. Dabei ist die gleichzeitige Einhaltung der gesetzlichen Datenschutzanforderungen essenziell und muss durch geeignete Maßnahmen, wie z. B. eine zuverlässige Deidentifizierung, sichergestellt werden [9, 10].

Konkrete Anforderungen an die Beschreibung und Kodierung von nachnutzbaren Daten werden in den sogenannten FAIR-Prinzipien beschrieben (FAIR steht als Akronym für Findable = auffindbar, Accessible = erreichbar, Interoperable = interoperabel, Reusable = nachnutzbar; [11]). Diese Anforderungen entlang des Datenlebenszyklus sind in den vergangenen Jahren zu einem internationalen Maßstab für wiederverwendbare Daten geworden. Eine Positionierung zu den FAIR-Kriterien wird bei Drittmittelanträgen in der Regel als Teil des Forschungsdatenmanagements (FDM) gefordert oder ist explizit Teil von Datenmanagementplänen. Die Europäische Kommission erwartet darüber hinaus die Stellungnahme zur Umsetzung dieser Maßnahmen in den Zwischenberichten. Hierdurch soll das Bewusstsein für die nachvollziehbare Bereitstellung von wissenschaftlichen Ergebnissen gestärkt und die Reproduzierbarkeit von Analyseergebnissen verbessert werden. Anstoß für die sehr dynamische Entwicklung im Gebiet des FDM waren sicherlich die sogenannte Reproduzierbarkeitskrise^2^ und die Digitalisierungsinitiativen des Bundes. In deren Folge sind in den Bundesländern Netzwerke für FDM entstanden, die die Aufgaben auf Landesebene organisieren und bundesweite Strategien mitentwickeln [12].

Verschiedene nationale Forschungsverbünde und Infrastrukturprojekte im Gesundheitswesen haben sich bereits klar zu den FAIR-Prinzipien positioniert: Sowohl die Infrastrukturen der MII als auch die der NUM operieren explizit auf Basis der FAIR-Prinzipien. Die MII setzt mit verschiedenen Maßnahmen die FAIR-Kriterien um. Die Auffindbarkeit wird beispielsweise durch das zentrale Deutsche Forschungsdatenportal für Gesundheit^3^ (FDPG) gewährleistet. Erreichbarkeit und Interoperabilität werden unter anderem durch den gemeinsamen modularen Kerndatensatz (KDS) erreicht, welcher die klinischen Daten aus den Primärsystemen über sogenannte ETL(extrahieren, transformieren, laden)-Prozesse in ein interoperables Format überführt und für die Sekundärnutzung bereitstellt [1]. Dabei legt der verwendete Datenaustauschstandard HL7 FHIR (Health Level 7 Fast Healthcare Interoperability Resources) nicht nur das Datenformat fest, was als syntaktische Interoperabilität bezeichnet wird, sondern definiert mithilfe von Terminologien wie der Systematisierten Nomenklatur der Medizin (SNOMED), der Logical Observation Identifiers Names and Codes (LOINC) und der International Classification of Diagnoses (ICD) auch die Bedeutung der Datenelemente und einzelnen Informationen. Dies wird als semantische Interoperabilität bezeichnet. Für die Nachnutzbarkeit wurden eine harmonisierte Patienteneinwilligung sowie vertragliche Rahmenbedingungen im sogenannten Teilnahmevertrag formuliert. Die Nationale Forschungsdateninfrastruktur für personenbezogene Gesundheitsdaten (NFDI4Health; [13]) entwickelt neben einem Metadatenschema (MDS) für epidemiologische und Gesundheitsstudien [14] mit den entsprechenden FHIR auch einen zentralen Study-Hub^4^ [15] und unterstützt somit die Auffindbarkeit und Exploration von Studieninformationen. Die FAIRe Bereitstellung von Daten sowie deren Nachnutzung waren 2 Kriterien bei der Erstellung des Basisdatensatzes für klinische Parameter in der Diabetes- und Stoffwechselforschung des Deutschen Zentrums für Diabetesforschung (DZD; [16]).

Im Idealfall können die aus den Daten gewonnenen Erkenntnisse wieder in die Versorgung zurückgespielt werden und dort zur verbesserten Behandlung von Patientinnen und Patienten beitragen [1]. Solche Feedbackmechanismen in die Klinik führen zunehmend zu einer engen Verzahnung von Versorgungs- und Forschungs-IT. Als Beispiel sei hier eine IT-Referenzarchitektur für Datenintegrationszentren aus dem SMITH(Smart Medical Technology for Healthcare; [17])-Konsortium genannt. Teil dieser Architektur ist eine klinische Domäne, die sämtliche Daten aus den klinischen Primärsystemen entgegennimmt und diese in den interoperablen, von der MII definierten KDS abbildet. Die klinische Domäne enthält also ein interoperables Abbild der Behandlungsdaten, mit dem z. B. klinische Entscheidungsunterstützungssysteme entwickelt und dann bei Erfolg direkt in der Versorgung verwendet werden können.

**Abb. 1:**
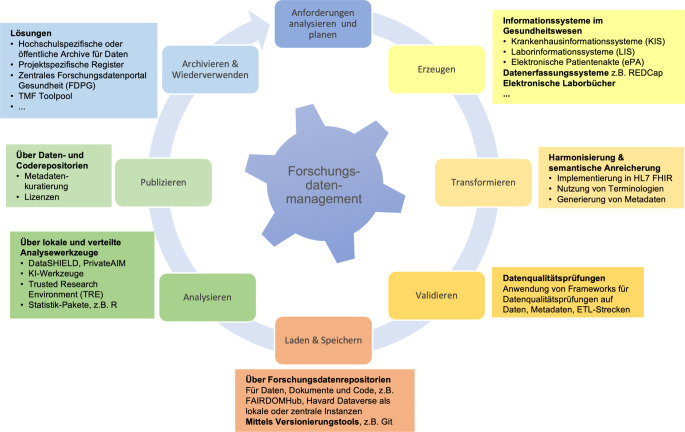
Konzepte und Werkzeuge für FAIRes Forschungsdatenmanagement entlang des Datenlebenszyklus. In der Gesundheitsforschung werden heterogene Daten in dynamischen Infrastrukturen unter Einhaltung gesetzlicher Vorgaben verwaltet. Dies erfordert eine gezielte Planung und Umsetzung einheitlicher Strategien für das Datenmanagement entlang des Datenlebenszyklus. Planung befasst sich mit vorausschauenden Aspekten zu Durchführung und Dokumentation zum Sammeln, Transformieren, Qualitätsanalyse, statistische Analyse und Reporting von Gesundheitsdaten sowie deren Archivierung. Zentral ist hierbei die Auswahl geeigneter Metadaten und Metadatenstandards in einer handhabbaren Granularität und hoher Qualität. Beispielhaft zeigt die Abbildung die Verarbeitung von Daten des MII-KDS mithilfe standardisierter ETL(extrahieren, transformieren, laden)-Entwicklungsprozesse entlang des Datenlebenszyklus^a^. Der Datenlebenszyklus beschreibt den Weg der (Wieder‑)Verwendung von Routinedaten von der Datensammlung bis zur endgültigen Analyse und Veröffentlichung unter Berücksichtigung (inter)nationaler Vorgaben, z. B. Datenschutz-Grundverordnung (DSGVO). Dabei erfolgt der Datentransfer mehrstufig mit dem Ziel, die Daten in interoperable Datenformate und -standards zu konvertieren. Methodisch gelingt dies unter Anwendung eines gezielten Datenmappings der Rohdatenwerte in einer temporären Speicherumgebung bzw. lokalen Data-Marts. *REDCap* Research Electronic Data Capture HL7; *FHIR* Healthcare Level 7 Fast Healthcare Interoperable Resources; *ETL* Extract, Transform, Load; *TMF* Technologie- und Methodenplattform für die vernetzte medizinische Forschung e. V.; *KI* Künstliche Intelligenz. (Quelle: eigene Abbildung). ^a^Gemäß https://forschungsdaten.info/themen/informieren-und-planen/datenlebenszyklus/, Zugegriffen: 26. Februar 2024

Nicht direkt in den FAIR-Kriterien adressiert sind die Aspekte Datenqualität und Reproduzierbarkeit. Wichtige Indikatoren für Datenqualität sind Vollständigkeit, Korrektheit und Konsistenz. Speziell für die Qualitätsprüfung der Forschungsdaten aus der klinischen Routine wurden verschiedene Systeme und Indikatoren entwickelt, die mehr oder weniger eng an den klinischen Routinesystemen gemessen werden [18, 19]. Hier ist ein Vorteil von HL7 FHIR, dass die beschriebenen Metadaten direkt in FHIR integriert werden können, wodurch in der Regel eine höhere Qualität der Daten erreicht wird. Reproduzierbarkeit erscheint in digitalen Anwendungen im Vergleich zu z. B. Laborexperimenten relativ einfach: Die gleichen Eingangsdaten sollen mit den gleichen Analysemethoden die gleichen Ergebnisse liefern. In der Realität können unterschiedliche Softwareversionen und unterschiedliche Hardware zu unterschiedlichen Ergebnissen führen. Reproduzierbarkeit erfordert deshalb neben der oben genannten Provenienz auch eine umfangreiche Dokumentation und idealerweise eine Archivierung der in der Analyse genutzten Software und Ausführungsumgebungen. Grundsätzlich kann dies durch die Anwendung der FAIR-Kriterien auf alle am Datenlebenszyklus beteiligten digitalen Objekte erreicht werden (Abb. 1).

## Wege zu FAIRen Forschungsergebnissen

Die FAIR Guiding Principles for Data Stewardship [11] wurden 2016 publiziert. Für auffindbare, erreichbare, interoperable und nachnutzbare Forschungsdaten werden in einem Kriterienkatalog verschiedene Anforderungen an die Daten spezifiziert (Tab. 1; [20–23]). Beispiele für die Umsetzung dieser Kriterien sind die genaue Beschreibung der Daten mit Metadaten, die Nutzung eines global einzigartigen, beständigen Identifier für die Auffindbarkeit (F1), die Nutzung von offenen Kommunikationsstandards für die Erreichbarkeit (A1), die Verwendung von FAIRen Vokabularien (I2), formalen, zugänglichen, gemeinsamen und allgemein anwendbaren Wissenssprachen und qualifizierten Referenzen zu anderen Daten für die Interoperabilität (I3) sowie die Angabe einer Nutzungslizenz (R1.1) und detaillierter Provenienzinformationen für die Nachnutzbarkeit (R1.2).

**Tab. 1:** Anforderungen an Gute Forschungsdaten gemäß FAIR-Kriterienkatalog [11] am Beispiel ausgewählter Gesundheits(meta)daten: Die Anforderungen werden klassifiziert nach der Verbesserung der Auffindbarkeit (Findability), Erreichbarkeit (Accessibility), Interoperabilität (Interoperability) und Nachnutzbarkeit (Reusability). Spalten 2 und 3 der Tabelle zeigen exemplarisch positive Beispiele aus aktuellen Forschungsdatensätzen ausgewählter nationaler Infrastruktur- und Verbundprojekte. Die Tabelle erhebt keinen Anspruch auf Vollständigkeit

FAIR-Kriterium	Beispiel	Erklärung
*Findable*		
F1: Datenpunkte und Metadaten besitzen einen global eindeutigen und dauerhaft gültigen Identifier	Jedes Element des NFDI4Health-MDS ist durch einen Identifier identifiziert	Jedes Element des NFDI4Health-MDS ist mit einem eindeutigen Identifier innerhalb des MDS-Namensraums versehen, der einem bestimmten Schema folgt. Dadurch kann auf alle Metadaten eindeutig verwiesen werden
F2: Die Daten sind mit reichhaltigen Metadaten annotiert	–	–
F3: Metadaten müssen eindeutig und explizit den Identifier der beschriebenen Daten enthalten	Die eindeutige Beschreibung der Metadaten des zentralen Study-Hubs der NFDI4Health ist im MDS spezifiziert [14]	Zur Referenzierbarkeit von Metadaten (z. B. wenn sie getrennt von den eigentlichen Datensätzen abgelegt werden) ist es notwendig, dass Metadaten eindeutig identifizierbar sind
F4: (Meta‑)Daten werden in einer durchsuchbaren Ressource registriert oder indiziert	Das FDPG bietet angemeldeten Nutzerinnen und Nutzern die Möglichkeit, verfügbare KDS-Daten über die angeschlossenen Datenintegrationszentren zu suchen	Eine Suche über Datenmodelle und deren Metadaten stellt sicher, dass Nutzerinnen und Nutzer darüber informiert werden, welche Daten über ein Repository bereitgestellt werden können und dass diese Daten auch gefunden werden
*Accessible*		
A1: (Meta‑)Daten sind anhand ihres Identifier über ein standardisiertes Kommunikationsprotokoll abrufbar	Die Datenpunkte des MII-KDS sind über eindeutige Identifier über https referenzierbar	Der diagnostische Laborbefund ist erreichbar über https://www.medizininformatik-initiative.de/fhir/core/modul-labor/StructureDefinition/DiagnosticReportLab
A1.1: Das Protokoll ist offen und universell implementierbar	Datenherausgabeprozess über das FDPG	Für die Datenherausgabe über das FDPG existiert ein protokollierter Standardprozess
A1.2: Das Protokoll unterstützt ein Authentifizierungs- und Autorisierungsverfahren, falls erforderlich	–	–
A2: Metadaten sind auch zugänglich, wenn die dazugehörigen Daten nicht verfügbar sind	Das Datenmodell des DZD-KDS ist über das MDM-Portal [12] öffentlich zugänglich, ohne dass die zugehörigen Daten verfügbar sind	Potenzielle Datennutzerinnen und -nutzer sowie Kooperationspartnerinnen und -partner können sich über das MDM bereits über die am DZD verfügbaren Datenpunkte informieren, ohne selbst Zugriff auf die Daten zu erhalten. Es besteht ebenso die Möglichkeit, Datenmodelle über Wissensgraphen miteinander zu verknüpfen, z. B. [20]
*Interoperable*		
I1: (Meta‑)Daten verwenden eine formale, zugängliche, gemeinsame und breit anwendbare Sprache zur Wissensdarstellung	Der MII-KDS ist im FHIR-Format implementiert (z. B. Modul Person, https://simplifier.net/MedizininformatikInitiative-ModulPerson/)	Die Verwendung von FHIR garantiert, dass Menschen (und Maschinen) die Daten lesen, (gleichartig) interpretieren und miteinander über verschiedene Anwendungssysteme hinweg austauschen können, z. B. über ein verteiltes Anfragesystem [21]
I2: (Meta‑)Daten nutzen Vokabulare, die den FAIR-Prinzipien folgen	Der MII-KDS nutzt LOINC zur Annotation der Laborparameter	LOINC selbst kann als FAIRes Vokabular betrachtet werden. Somit wird sichergestellt, dass die Vokabulare, die genutzt werden, um die Daten zu beschreiben, selber zugänglich und verständlich sind
I3: (Meta‑)Daten enthalten qualifizierte Referenzen zu anderen (Meta‑)Daten	Metadaten im HL7-FHIR-Standard verlinken auf weitere (FHIR-)Ressourcen und kontrollierte Vokabulare	Beispielsweise verweisen Abrechnungsdaten aus dem MII-KDS auf ICD-10-GM-Kodierungen (erforderlich laut §§ 301, 295, 5. Sozialgesetzbuch). Eine Vielzahl solcher qualifizierten Referenzen auf andere Vokabulare ist notwendig, um ein umfängliches Bild der Daten zu erhalten [3]
*Reusable*		
R1: (Meta‑)Daten sind detailliert beschrieben und enthalten präzise, relevante Attribute	–	–
R1.1: (Meta‑)Daten enthalten eine eindeutige zugreifbare Angabe einer Nutzungslizenz	License DZD-KDS: Creative Commons BY-SA 4.0	Der DZD-KDS ist mit einer offenen Standardlizenz der Creative Commons versehen. Die Lizenz ist Teil der Metadaten. Sie klärt eindeutig die Bedingungen zur Nachnutzung eines Datensatzes [16]
R1.2: (Meta‑)Daten enthalten detaillierte Provenienzinformationen	–	Wünschenswert wäre die Implementierung eines W3C-Standards für Provenienzinformation über die KDS-Elemente [22]
R1.3: (Meta‑)Daten entsprechen fachgebietsrelevanten Standards	Repräsentation des MII-KDS als FHIR-Ressourcen	Die Verwendung des internationalen HL7-FHIR-Standards erhöht die Interoperabilität der MII-Ressourcen mit Software, aber beispielsweise auch mit dem MDS der NFDI4Health, welches ebenfalls mit FHIR-Ressourcen^a^ abgebildet wurde [23]

*NFDI4Health* Nationale Forschungsdateninfrastruktur für personenbezogene Gesundheitsdaten, *MDS* Metadatenschema, *FDPG* Forschungsdatenportal für Gesundheit, *KDS* Kerndatensatz, *DZD* Deutsches Zentrum für Diabetesforschung, *MDM* Medical Data Model, *MII* Medizininformatik-Initiative, *FHIR* Fast Healthcare Interoperability Resources, *LOINC* Logical Observation Identifiers Names and Codes, *HL7* Health Level 7, *ICD-10-GM* International Classification of Diagnoses 10. Revision German Modification, *W3C* World Wide Web Consortium

^a^https://simplifier.net/NFDI4Health-Metadata-Schema/, Zugegriffen: 26. Februar 2024

Sobald für eine Ressource entschieden wird, die FAIR-Prinzipien einzuhalten, sollte eine erste Evaluation erfolgen, um den aktuellen Grad der FAIRness festzustellen (sog. FAIR-Assessment) und dann im zweiten Schritt die Priorisierung für Verbesserungsschritte durchzuführen (FAIRification). Seit 2016 wurde eine Reihe von Werkzeugen und Richtlinien für beide Schritte entwickelt, sowohl für Daten als auch Infrastrukturkomponenten und basierend auf den unterschiedlichen, domänenspezifischen Interpretationen der FAIR-Prinzipien.

### Feststellung der FAIRness einer Ressource: FAIR-Assessment

Ziel der 2013 gegründeten internationalen Research Data Alliance (RDA) ist es, den Datenaustausch über Technologien, Disziplinen und Länder hinweg zu ermöglichen [24]. Als eine der ersten Initiativen hat die RDA bereits 2019 eine Arbeitsgruppe zur Entwicklung eines Bewertungsschemas für die FAIRness von Daten gegründet. Dieses Schema wurde später als „FAIR Data Maturity Model“ [25] bekannt und gilt heute als ein zentraler Bewertungskatalog in vielen Wissenschaftsbereichen [26]. Ergebnis der Arbeit ist eine Liste von domänenunabhängigen FAIRness-Indikatoren mit einem zugehörigen Reifegradmodell. Basierend darauf entstanden verschiedene Richtlinien und Checklisten zur Implementierung der FAIR-Indikatoren [27]. Der Grad der FAIRness kann pro Indikator auf einer 5‑stufigen Skala oder durch Zuweisung einer Ja/Nein-Bewertung angegeben werden („not applicable bis fully implemented“; [26]). Darüber hinaus unterstützen diverse Werkzeuge das FAIR-Assessment, beispielsweise manuelle Fragebögen, Checklisten oder automatisierte Tests.

**Abb. 2:**
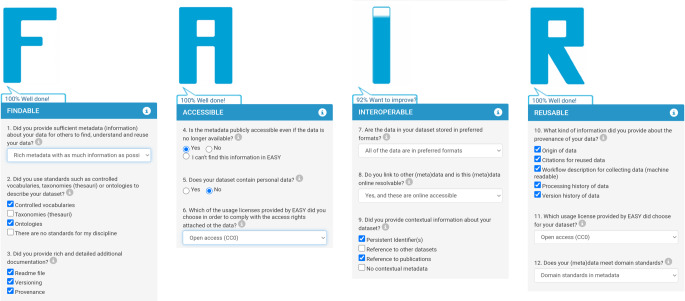
Ergebnisse des FAIR-Assessments des Basisdatensatzes des Deutschen Zentrums für Diabetesforschung (DZD) nach Umsetzung der Empfehlungen des SATIFYD-Tools. Im Rahmen eines strukturierten FAIRification-Assessments wurden folgende Schritte auf die ursprüngliche Version des Kerndatensatzes (KDS) angewendet: Zuweisung einer dauerhaften Kennung sowohl für die Daten als auch für die Metadaten, Versionierung, Konvertierung in das empfohlene Format für Tabellenkalkulationen, Annotation der darin enthaltenen Parameter mit dem Unified Medical Language System (UMLS), Lizenzierung des Datensatzes, Indizierung der Metadaten als eine durchsuchbare Ressource, die den Datensatz großzügig mit Metadaten anreichert und Herkunftsinformationen bereitstellt. Diese Maßnahmen führten zu einem FAIRification-Score von 98 % im Online-Assessment-Tool SATIFYD^a^ (Quelle: eigene Abbildung). ^a^https://satifyd.dans.knaw.nl/, Zugegriffen: 26. Februar 2024

Manuelle FAIR-Bewertungsmethoden in der Form von Self-Assessments bieten eine sehr einfache und schnelle Möglichkeit, die FAIRness eines Forschungsobjekts zu bewerten, werden jedoch in der Literatur als schwerfällig beschrieben [28]. Ein Beispiel für das FAIR-Assessment des DZD-Basisdatensatzes mit SATIFYD (Self-Assessment Tool to Improve the FAIRness of Your Dataset) zeigt Abb. 2.

Halbautomatische Methoden ermöglichen es, die Bewertung teilweise automatisch vorzunehmen und die Ergebnisse anschließend zu korrigieren, zu verfeinern und zu vervollständigen [29]. Automatisierte Bewertungen werden in der Regel über Webanwendungen und entsprechende Schnittstellen (APIs) zugänglich gemacht. Das zu bewertende Forschungsobjekt wird in der Regel über einen Uniform Resource Identifier (URI) oder einen Digital Object Identifier (DOI) bereitgestellt.

Für das FAIR-Assessment im medizinischen und Gesundheitsbereich spielen die semiautomatischen Verfahren eine entscheidende Rolle, da insbesondere die Einschätzung des Informationsgehalts von Metadaten eine manuelle Bewertung erfordert. Andere Aspekte können sehr gut automatisiert bewertet werden, beispielsweise die Feststellung der Maschinenlesbarkeit [30]. Der Einsatz halbautomatischer Bewertungstools erfordert jedoch Übung im Umgang und Kenntnisse darüber, wie sich die jeweiligen Indikatoren auf die Daten und FDM-Praktiken auswirken [26]. Vorschläge für entsprechende Werkzeuge sind in Tab. 2 zusammengefasst [31–38].

**Tab. 2:** Auswahl an FAIR-Assessment-Werkzeugen. Es wird unterschieden nach manuellen (m), semiautomatischen (s) und automatischen (a) Werkzeugen für das FAIRness-Assessment von Ressourcen, insbesondere (Meta‑)Datenspezifikationen

Werkzeug	Kurzbeschreibung	Typ
FAIR Data Maturity Model (FDMM; [26])	Definition einer Menge von Indikatoren für FAIRness mit verschiedenen Implementierungsstufen	m
SHARC [31]	Assessment-Tool basierend auf den Regeln und Prinzipien der Web Content Accessibility Guidelines^a^	m
Ontology FAIRness Evaluator (O’FAIRe; [32])	Werkzeug zur automatischen Bewertung der FAIRness einer semantischen Ressource oder Ontologie	a
FOOPS [33]!	Online-Dienst zur Bewertung der Konformität von Vokabularen oder Ontologien mit den FAIR-Grundsätzen	a
F‑UJI [34]	Automatisches Online-Assessment-Tool basierend auf von FAIRsFAIR entwickelten Metriken	a
10 simple rules for making a vocabulary FAIR [35]	Anleitung mit 10 Schritten um ein Vokabular FAIR zu machen	m
ARDC FAIR self-assessment [36]	Kurzer Fragebogen mit hilfreichen Erklärungen für jede Frage	m
FAIRshake [37]	Assessment-Tool für digitale Objekte	s
FAIR-Aware [38]	Fragebogen mit 10 Fragen und hilfreichen Erklärungen	m

^a^https://www.w3.org/TR/WCAG20/, Zugegriffen: 26. Februar 2024

### Workflows zur Verbesserung der FAIRness: FAIRification

Sobald ein FAIR-Assessment für einen Datensatz vorliegt, kann ein strukturierter Prozess zur Verbesserung der FAIRness gestartet werden. Ziel dieser FAIRification ist es, in den 4 Teilbereichen (F, A, I, R) eine verbesserte Wertung (Score) zu erhalten. Hierbei wird in der Regel nicht auf die 100-prozentige Erfüllung aller Kriterien abgezielt, sondern es werden die projektspezifischen Anforderungen analysiert und bestimmte Kriterien entsprechend priorisiert. Beispielsweise sollte ein Metadatenschema vor allem zugreifbar und interoperabel sein, ein Datensatz eher auffindbar und interoperabel, ein Provenienzmodell vor allem nachnutzbar. Interoperabilität hat im Allgemeinen einen besonderen Stellenwert für die Wiederverwendung von Daten im Gesundheitswesen. Im medizinischen Kontext beschreibt HL7 FHIR Datenformate und Elemente als sogenannte Ressourcen und bietet Schnittstellen an, um diese auszutauschen. Die Vorteile der etablierten HL7-Standardproduktlinien Version 2, Version 3 und Clinical Document Architecture (CDA) werden dabei mit jenen aktueller Webstandards kombiniert. Ein starker Fokus liegt dabei auf einer einfachen Implementierbarkeit, um den Datenaustausch zwischen Softwaresystemen im Gesundheitswesen zu befördern.

Auch FAIRification wird durch verschiedene Werkzeuge unterstützt, die entweder generisch oder domänenspezifisch gehalten sind [39]. Speziell an die Anforderungen von Gesundheitsforschungsdaten angepasste Workflows berücksichtigen vor allem die technischen, ethischen und rechtlichen Anforderungen [40]. Das FAIRplus-Konsortium^5^ hat hierfür ein umfassendes Rahmenwerk entwickelt [41], welches frei von spezifischen Implementierungslösungen oder -methoden verwendbar ist. Es besteht aus 4 verschiedenen Phasen: einer Zieldefinitionsphase, einer anfänglichen Projektprüfungsphase, einer iterativen zyklischen FAIRifizierungsphase und einer Post-FAIRifizierungsüberprüfung. Ein weiterer FAIRification-Workflow für Gesundheitsdaten wurde vom FAIR4Health-Projekt entwickelt und evaluiert [40, 42].

Das DZD strebt eine FAIRe Datenverwaltung auf Basis des DZD-KDS an, um die Nutzung der Daten innerhalb des DZD und der breiteren Diabetes-Forschungsgemeinschaft zu unterstützen. Im Rahmen eines strukturierten, begleitenden FAIRification-Prozesses wurde beispielsweise der DZD-KDS in der Entwicklungsphase bereits mit Metadaten angereichert und im Medical-data-model(MDM)-Portal registriert. Er steht in mehreren maschinen- und menschenlesbaren Formaten zur Verfügung. Mit einer offenen Lizenz trägt er zur Standardisierung in der Erfassung von Stoffwechselparametern in der klinischen Forschung bei. Das Ergebnis des manuellen FAIR-Assessments nach der FAIRification ist in Abb. 2 abgebildet. Weitere Beispiele für bereits erfolgte FAIR-Assessments mit partieller FAIRification sind das NFDI4Health-Metadatenschema, die LOINC-Kodierung innerhalb der SHIP(Study of Health in Pomerania)-Studie, die Datensätze der CODEX+(COVID-19 Data Exchange Platform)-Projekte oder biomedizinische Simulationsmodelle.

## FAIR betrifft alle Bausteine des Forschungsdatenmanagements

Die FAIR-Prinzipien werden häufig im Kontext von (Forschungs‑)Daten betrachtet, jedoch wird bereits in der Originalpublikation darauf hingewiesen, dass sie grundsätzlich auf alle digitalen Artefakte in einem Forschungsprozess angewendet werden sollen, also auch auf Software, Analyseskripte oder auch Ausführungsumgebungen. Im Kontext der MII betrifft dies z. B. die Implementierung der ETL-Prozesse in den Datenintegrationszentren, aber man kann dies grundsätzlich auch auf die Informations- und Entscheidungsunterstützungssysteme der Krankenversorgung beziehen, die sowohl als Quell- als auch als Zielsysteme insbesondere in der versorgungsnahen Forschung dienen können. Im Prinzip lassen sich viele der oben genannten FAIR-Kriterien direkt auf Software und Ausführungsumgebungen anwenden, wie z. B. die Bereitstellung von Software über Plattformen wie codeberg, mit eindeutigen Identifier für verwendete Softwareversionen oder die Bereitstellung von Ausführungsumgebungen als virtuelle Maschinen oder Container [43]. Allerdings existieren bisher kaum Metadatenstandards für die Beschreibung von Software und Ausführungsumgebungen [44, 45]. Auch für die Nutzbarkeit von Versorgungsdaten, beispielsweise in KI-Anwendungen, können die FAIR-Kriterien herangezogen werden, um die Reproduzierbarkeit von Modellen zu erhöhen. Die Abdeckung der FAIR-Kriterien in Guidelines für KI-Modellentwicklung variiert hierbei stark – weitere Forschung ist auf diesem Gebiet erforderlich [46].

Die gemeinsame (Nach‑)Nutzung von Ausführungsumgebungen als sogenannte vertrauenswürdige Forschungsumgebungen (Trusted Research Environments; [47]), die die Herausgabe von Gesundheitsdaten vermeiden, geht darüber hinaus und erfordert insbesondere Maßnahmen der sicheren Nutzung durch verschiedene Anwenderinnen und Anwender. Auch solche Umgebungen können im Rahmen von FAIR-Kriterien beschrieben werden, so z. B. Interoperabilität der Prozesse und Nutzungsdaten, definierte Nutzungsbedingungen und Verwendung von offenen Kommunikationsstandards [46]. Andere Anforderungen wie Skalierbarkeit und Sicherstellung von Vertrauenswürdigkeit gehen darüber hinaus.

## Kosten vs. Nutzen von FAIRification

Eine der größten Herausforderungen bei dem Bestreben, Daten und Infrastrukturen FAIR zu gestalten – und vielleicht der digitalen Transformation insgesamt – besteht darin, dass diejenigen, die von der Nachnutzung der Daten profitieren, oftmals andere Personen sind als diejenigen, die sie erheben und aufbereiten. Der Konflikt zwischen diesen beiden Interessengruppen wird bei Gesundheitsdaten sehr deutlich. Quellsysteme wie Krankenhausinformationssysteme und elektronische Patientenakten basieren meist auf Freitext, der nur sehr begrenzt strukturiert ist und wichtige Datenelemente nur teilweise erfasst. Dem medizinischen Fachpersonal steht nur eine begrenzte Zeit für die Dateneingabe zur Verfügung. Andererseits können geringe Qualität, unvollständige und unstrukturierte Daten im Quellsystem nicht durch FAIRification korrigiert werden. Um Daten effektiv und in der Breite wiederverwenden zu können, wie z. B. durch Forschende, aber auch durch medizinisches Fachpersonal, Patientinnen, Patienten und Industrie, muss in die Quellsysteme investiert werden, um die Datenqualität und FAIRness am Ort der Datenerhebung zu verbessern.

Unter diesem Gesichtspunkt ist es natürlich wichtig, die Perspektive der Datenproduzentinnen und -produzenten zu berücksichtigen. Im Gesundheitswesen ist dies üblicherweise das medizinisch-pflegerische Fachpersonal. Es muss diskutiert werden, in welchem Verhältnis die Kosten für die FAIRe Datenbereitstellung zu den jeweiligen Vorteilen stehen. Kosten können beispielsweise durch eine bessere Digitalisierung gesenkt werden, aber auch durch Prozessoptimierungen und Reduktion doppelter und grundsätzliche Vereinfachung von Dateneingaben. Weiterhin können die Vorteile guter Daten direkt an die Datenerzeuger zurückgespielt werden, beispielsweise indem offene Werkzeuge bereitgestellt werden, um Patientendaten zu analysieren, mit ähnlichen Patientinnen und Patienten (auch aus anderen Einrichtungen) zu vergleichen und mit visueller Unterstützung die Daten der jeweiligen Gesundheitseinrichtung zu analysieren oder zu explorieren. Auf diese Weise werden die Datenproduzentinnen und -produzenten zeitnah zu denjenigen, die von den aufbereiteten, semantisch angereicherten FAIRen Daten profitieren.

## Anknüpfung an nationale und internationale Initiativen

Viele europäische Nachbarländer haben in den letzten Jahren in die Entwicklung eines nationalen Rahmens für semantische Interoperabilität von Gesundheitsdaten investiert. So wurden Konzepte für eine umfassende Datenanreicherung entwickelt, um die Datenanalyse zu vereinfachen sowie die korrekte Interpretierbarkeit der Daten bei deren Weiterverwendung in verschiedenen Kontexten zu erhalten. Dies zumeist mit dem Ziel, nachhaltige und skalierbare Prozesse und Strukturen zu entwickeln, die eine zeit- und kosteneffiziente Bereitstellung von standardisierten, FAIRen Gesundheitsdaten für die Primär- und Sekundärnutzung gewährleisten.

Die nationalen Initiativen, die vielerorts länderübergreifend in engem Austausch stehen, entwickeln zumeist individuelle, unabhängige Informationsmodelle, welche die Bereitstellung harmonisierter Daten für Forschungszwecke ermöglichen. Dabei beziehen sich alle auf international etablierte Kodiersysteme, Ontologien und Terminologien, wie beispielsweise ICD, SNOMED oder LOINC. Die nationalen Forschungsdateninfrastrukturen (NFDIs) in Deutschland tätigen wichtige fachübergreifende Investitionen in die Implementierung der FAIR-Kriterien. Im Gesundheitsbereich sind hierbei das Deutsche Humangenom-Phänomarchiv^6^ (GHGA), die NFDI4Immuno^7^ und NFDI4Health^8^ als Beispiele zu nennen.

Selbstverständlich existieren auch länderspezifische Standards, die aufgrund der Kodierungsprozesse für die Vergütung von Gesundheitsleistungen in den Krankenhäusern verwendet und im Rahmen der Initiativen entsprechend genutzt werden. Während der modulare MII-KDS die Struktur, das Format und die semantische Annotation der MII-Daten auf der Grundlage von HL7 FHIR beschreibt, setzt beispielsweise die Swiss-Personalized-Health-Network-(SPHN-)Initiative^9^ mit ihrer Interoperabilitätsstrategie auf die semantische Darstellung von Gesundheitsdaten in einem „Knowledge Graphen“, unter Anwendung der „Semantic Web Technologie“ und stringenter Umsetzung der FAIR-Prinzipien, um die gemeinsame Nutzung und nahtlose Integration unterschiedlicher gesundheitsbezogener Daten zu erleichtern [48]. Ein konkretes Beispiel für die länderübergreifende FAIRification von Gesundheitsdaten ist die gemeinsame Arbeit an einem Provenienzmodell für die KDS der MII und SPHN im Rahmen eines Community-getriebenen Projekts^10^ (MInimal Requirements for Automated Provenance Information Enrichment [MIRAPIE]).

Die Interoperabilitätsstrategie der niederländischen Initiative „Health-RI“ befindet sich derzeit noch in der Planung und soll in einem sogenannten Blütenmodell organisiert werden, bei welchem der KDS unter Nutzung international anerkannter Datenstandards zentral definiert wird und die verschiedenen Blütenblätter individuelle Projekt- oder Bereichsschemata darstellen. Das FAIR-Datenimplementierungsteam von Health-RI entwickelt zusammen mit den Netzwerkpartnern einen Ansatz für die FAIRe Datenimplementierung, d. h. die Bereitstellung von Datensätzen und zugehörigen (reichhaltigen) Metadaten in allen angeschlossenen Organisationen, welche Daten verwalten. Ziel ist es, Prozesse zur FAIRification so früh wie möglich im Datenbereitstellungsprozess anzugehen und weitestgehend zu automatisieren. In mehreren skandinavischen Ländern, aber beispielsweise auch in Katalonien kommt openEHR^11^ zum Einsatz. openEHR bietet die Möglichkeit, eine FAIR-konforme klinische Datenressource aufzubauen, indem die openEHR-Spezifikationen in Kombination mit einigen Ad-hoc-Einsatzkonfigurationen angewendet werden [49].

Die nationalen und länderübergreifenden Dateninitiativen der europäischen Länder spielen eine entscheidende Rolle bei der Förderung der Weiterverwendung klinischer Gesundheitsdaten sowie biologischer Humandaten im Rahmen des Europäischen Gesundheitsdatenraums^12^ (EHDS). Die Vorarbeiten dieser Initiativen mit Blick auf FAIRe Gesundheitsdaten erfordern, dass auch auf europäischer Ebene eine harmonisierte und interoperable Dateninfrastruktur geschaffen wird, die den Austausch und die Weiterverwendung von Gesundheitsdaten für die Forschung erleichtert. Auf internationaler Ebene spielt die Interaktion mit Observational Health Data Sciences and Informatics (OHDSI) eine zunehmend wichtige Rolle. Die OHDSI-Community entwickelt Methoden zur Analyse von Beobachtungsdaten und wendet diese in konkreten Projekten auf große Datenmengen an. Darüber hinaus fördert OHDSI die offene Zusammenarbeit und den Austausch von Ideen, Methoden und Ergebnissen, beispielsweise durch Code-Repositorien, Tutorien und Workshops. Dies erleichtert die Interoperabilität und die Anwendung der FAIR-Prinzipien, indem es eine gemeinsame Struktur für verschiedene Arten von Gesundheitsdaten bereitstellt. In der MII und dem NUM (Projekt CODEX+) wurden bereits OHDSI-basierte Pakete entwickelt und bereitgestellt, die es den Uniklinika ermöglichen, an internationalen Studien teilzunehmen.

## Fazit

Forschung an Gesundheitsdaten führt zu neuen medizinischen Erkenntnissen, unterstützt Therapieentscheidungen, trägt zur Entwicklung neuartiger technischer und KI-basierter Entwicklungen für Datenerhebung und Datenanalysen bei und verbessert somit die Patientenversorgung. Hierbei sind viele Forschungsfragen auf nationaler und internationaler Ebene im Verbund zu adressieren. Das Teilen qualitätsgesicherter, vollständiger und gut dokumentierter Daten aus Versorgung und Forschung ist hierbei essenziell. Dies umfasst auch zunehmend genetische Daten und von Patientinnen und Patienten bereitgestellte Informationen.

Voraussetzung für alle Datennutzungsprozesse sind gute Planung und kontinuierliches Monitoring. Ein modernes FDM erhöht die Datenqualität, macht Ergebnisse transparent und unterstützt somit gute Forschung. Die FAIR-Prinzipien gelten dabei als ein De-facto-Standard für das FDM, denn sie schaffen insbesondere in Kombination mit Provenienzmodellen, Guter Wissenschaftlicher Praxis und der Umsetzung von Prinzipen für gute Software und Forschungsinfrastrukturen Vertrauen. Sie motivieren somit zur Nachnutzung vorhandener Daten.

Ein Grundmaß an FAIRness sollte für jedes Datenprojekt im Gesundheitssektor das Ziel sein, eine vollständige FAIRification ist wahrscheinlich nur in wenigen Projekten von praktischer Relevanz. Es ist daher essenziell, dass sich Forschende mit der Planung eines zielgerichteten FDM auseinandersetzen, insbesondere aber auch die Datenerhebenden in den Prozess einbezogen werden und Kompetenzen und Ressourcen entsprechend deren zentraler Rolle an den Datenquellen berücksichtigt werden. Neben der Nutzung an den Forschungseinrichtungen selbst, beispielsweise die Inanspruchnahme von Beratungsangeboten durch Data Stewards oder Weiterbildungen an Graduiertenakademien und Datenintegrationszentren, stehen auch auf nationaler und internationaler Ebene zahlreiche Angebote und Handbücher zur Verfügung. Beispielhaft seien die Landesinitiativen FDM^13^, die NFDI4Health oder Initiativen auf europäischer Ebene (ELIXIR^14^, Research Data Alliance) genannt.

Forschende sollten im Rahmen der gesetzlichen Möglichkeiten die zur Publikation gehörigen Datensätze und Metadaten publizieren. Wie die Daten nachgenutzt werden und in welcher Form die Datenproduzenten hierbei mit einbezogen werden sollen, klärt die Lizenzierung, deren Angabe Teil der FAIR-Anforderungen ist. Vertrauenswürdige, domänenspezifische Repositorien, wie beispielsweise der zentrale Study-Hub der NFDI4Health oder das FDPG für Routinedaten mit Broad Consent, stellen die publizierten Daten und Metadaten anschließend über koordinierte Prozesse für die Nachnutzung zur Verfügung. Die derartige Nachnutzung von Forschungsdaten garantiert in der Regel eine hohe Datenqualität und reduziert die Aufwände für Personal, IT-Infrastruktur und Zeit gegenüber der Erhebung von Primärdaten. Zu bedenken gibt es hierbei jedoch, dass die Datenqualität in den primären Erhebungssystemen bereits maßgeblich über die Qualität der daraus abgeleiteten Forschungsdaten bestimmt – somit also auch ein Umdenken in der Routinedatenerhebung erfolgen muss.

Gesundheitsdaten werden in ganz Europa auf unterschiedliche Weise erhoben. Nur wenn Gesundheitsdaten FAIR sind, können sie neben der lokalen und nationalen Nutzung auch an internationale Netzwerke anknüpfen. So wurden beispielsweise im Rahmen des EHDS Standards festgelegt, die sicherstellen sollen, dass Daten grenzüberschreitend für Forschung und Entscheidungsfindung genutzt werden können.^15^

## Footnotes

1 https://num-compass.science/, Zugegriffen: 26. Februar 2024.

2 Die sogenannte Reproduzierbarkeitskrise (Replikationskrise, Krise der Reproduzierbarkeit) bezeichnet die seit 2011 mehrfach systematisch belegte Beobachtung, dass publizierte Erkenntnisse durch die Wiederholung der beschriebenen Studien und Experimente nicht nachvollzogen werden können.

3 https://forschen-fuer-gesundheit.de/, Zugegriffen: 26. Februar 2024.

4 https://csh.nfdi4health.de, Zugegriffen: 26. Februar 2024.

5 https://fairplus-project.eu, Zugegriffen: 26. Februar 2024.

6 https://www.ghga.de/, Zugegriffen: 26. Februar 2024.

7 https://www.nfdi4immuno.de/, Zugegriffen: 26. Februar 2024.

8 https://www.nfdi4health.de/, Zugegriffen: 26. Februar 2024.

9 https://www.sphn.ch, Zugegriffen: 26. Februar 2024.

10 https://codeberg.org/MIRAPIE/, Zugegriffen: 26. Februar 2024.

11 https://www.openehr.org/, Zugegriffen: 26. Februar 2024.

12 https://www.european-health-data-space.com, Zugegriffen: 26. Februar 2024.

13 https://forschungsdaten.info/fdm-im-deutschsprachigen-raum, Zugegriffen: 26. Februar 2024.

14 https://elixir-europe.org/, Zugegriffen: 26. Februar 2024.

15 https://tehdas.eu/app/uploads/2022/02/tehdas-identification-of-relevant-standards-and-data-models-for-semantic-harmonization-2022-02-03.pdf, Zugegriffen: 26. Februar 2024.
